# (In)determinacy in Woody Plants: Limits and Opportunities for Timing Growth in a Changing Climate

**DOI:** 10.1111/ele.70435

**Published:** 2026-06-24

**Authors:** Frederik Baumgarten, Sally Aitken, Yann Vitasse, Robert D. Guy, EM Wolkovich

**Affiliations:** ^1^ Department of Forest and Conservation Sciences, Faculty of Forestry University of British Columbia Vancouver British Columbia Canada; ^2^ Department of Environmental Sciences‐Botany University of Basel Basel Switzerland; ^3^ Swiss Federal Institute for Forest, Snow and Landscape Research WSL Birmensdorf Switzerland

**Keywords:** carbon sequestration, drought, forest productivity, genetic programming, growing season length, indeterminate growers, phenotypic plasticity, plant growth, shoot extension, tree phenology

## Abstract

When and how much plants grow under environmental constraints are fundamental questions in biology and increasingly important for predicting biomass production and carbon sequestration under climate change. While temperature and water availability directly regulate plant growth, the timing and rate of growth are also shaped by internal developmental programming, though this is rarely considered in predictions of tree growth responses to future climates. Here, we revisit a concept central to this internal programming—(in)determinacy. Focusing on woody plants, we define it as the extent to which annual shoot growth is deployed from preformed organs versus produced de novo during the current season. We argue that this trait is best understood as a continuum and that it can help explain contrasting growth responses in a changing climate. More determinate species concentrate growth within a narrow seasonal window, which may reduce exposure to late‐season stress but also limit their ability to exploit longer growing seasons. More indeterminate species retain greater flexibility to extend or resume growth when conditions remain favourable, which may be advantageous under climate change, but this same flexibility may also increase exposure to frost, drought and incomplete tissue maturation. Because primary shoot growth also shapes canopy development and is linked to other growth processes, variation in (in)determinacy could help explain broader differences in whole‐plant performance, carbon gain and species responses to climate change.

## Graphical abstract

Tree growth is shaped not only by temperature and water availability, but also by intrinsic developmental programming that constrains when and how long meristems remain active. We use (in)determinacy—the continuum from fixed, preformed seasonal growth to more flexible, ongoing organ production—to predict how species will differ in risk exposure, recovery and their ability to benefit from longer growing seasons. Accounting for this continuum should improve predictions of forest productivity and carbon sequestration under climate change.

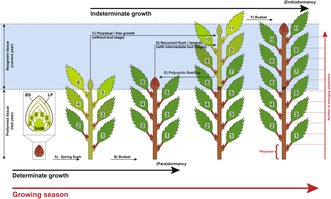

## 1 Introduction

Investing the right amount of resources in growth at the right time is of critical importance to the survival and fitness of any living organism. The topic of growth strategies and habits has a long history in science, spanning the fields of genetics, genomics, physiology and ecology across the animal and plant kingdoms (Stearns 1998). Central to this discussion is the concept of determinacy, or more broadly (in)determinacy, which describes the extent to which growth is fixed or remains open over time. At its broadest, determinacy has been used to classify organisms according to whether growth ceases at maturity or continues throughout life; in this sense, plants are often described as indeterminate growers because they can continue adding biomass throughout their lives (Ejsmond et al. 2010; Karkach 2006). In plants, however, the concept has also been applied at finer spatial and temporal scales, from single cells and organs to whole shoots and individuals and from a single season to an entire lifetime (McDaniel 1992; Karkach 2006).

In this Perspective, we focus on this narrower developmental meaning. In woody perennials, organs can develop from preformed tissue (e.g., primordia in overwintering buds), form *de novo*, or both, depending on species and life form. While these underlying developmental and meristematic constraints apply broadly across the plant kingdom, they play a particularly central role in the life cycle of woody perennials, especially trees growing in extra‐tropical regions, which must balance growth, survival and reproduction over long lifespans in highly seasonal environments. As we use it here, (in)determinacy in woody perennials refers to the ability to:

- preform organs in 1 year as a future investment, which is deployed rapidly in the following (or later) spring with no further primary shoot growth during that season (determinate strategy);
- maintain continuous or episodic meristem activity by forming new shoot tissue during the current growing season (indeterminate strategy).

In this Perspective, we revisit classical concepts of determinacy as they relate to shoot growth in woody perennials, place them in the context of climate change and explore to what extent related developmental principles may also inform growth processes in other meristematic tissues. Although we focus on woody perennials, the concept of (in)determinacy is broadly relevant across vascular plants, including herbaceous species; trees provide a particularly informative model system because the ecological and climatic consequences of growth timing are especially pronounced in long‐lived woody plants. To provide context, we first outline the fundamental limitations to plant growth in extra‐tropical regions by linking external environmental drivers with internal growth control mechanisms. In this context, our aim is to provide a conceptual and methodological basis for analysing (in)determinacy as a trait relevant to plant responses to climatic variability and change, to clarify how the concept can be defined and measured and to identify key research gaps and directions for future comparative and trait‐based analyses.

## 2 Seasonality of Temperature, Soil Moisture and Light

The further one travels from the equator toward the poles, the more strongly plants are confined to a shrinking temporal window of opportunity for growth set primarily by low temperatures. Below c. 5°C, metabolic activity slows to an extent where growth and development largely cease (Schenker et al. 2014; Rossi et al. 2008; Körner 2008). More importantly, sub‐freezing temperatures can cause severe damage to plant tissue if they occur at vulnerable developmental stages, for example, during or after leaf unfolding, prior to fruit maturation, or before cold acclimation in fall (Sakai and Larcher 1987; Baumgarten et al. 2023). While annual plant species accommodate their entire life cycle within this window, perennial (woody) species of extra‐tropical regions are forced to partition their growth phase seasonally, with periods of activity alternating with periods of dormancy (Delpierre et al. 2016). This is referred to as intermittent or rhythmic (as opposed to continuous) growth.

**FIGURE 1:**
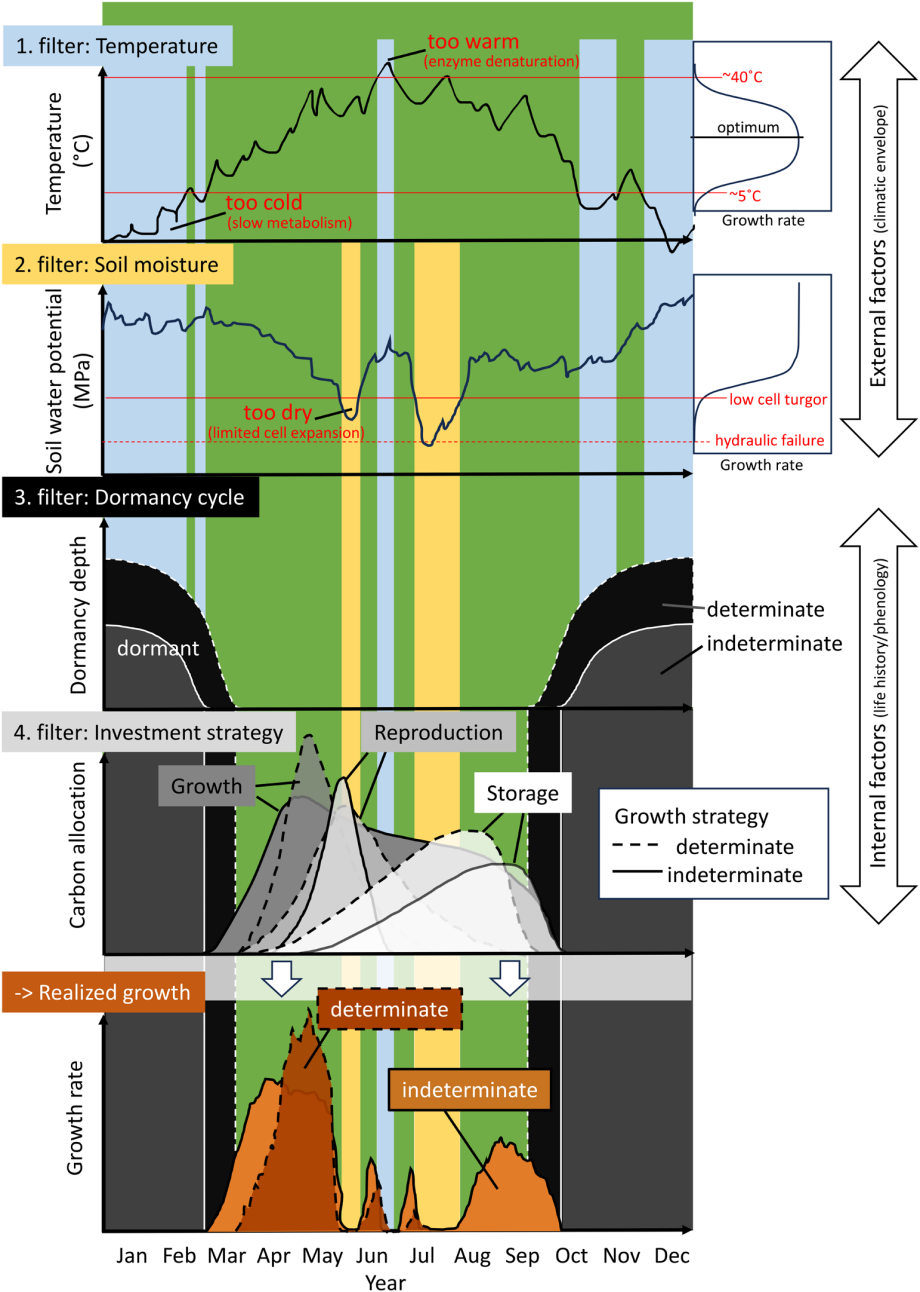
Schematic overview of the discrepancy between the potential growing season and the effectively realized period of vegetative growth (shoot and possibly cambial growth). Green indicates the potential growing season, whereas overlaid coloured areas represent portions of this period that are constrained by different external and internal filters. Environmental factors, such as temperature and soil moisture, can narrow the window of opportunity for vegetative growth when conditions fall outside growth‐promoting thresholds (potential light and photoperiodic constraints are not shown). Species‐specific phenology can impose an additional filter through dormancy and through prioritization of other developmental processes (e.g., reproduction and storage). The degree of (in)determinacy is illustrated here as two extremes, although we propose that this trait is better understood as continuous.

High temperatures can directly inhibit growth by exceeding species‐specific physiological thresholds (O'Sullivan et al. 2017). In addition, increased evaporative demand and reduced precipitation can lower soil moisture, leading to water stress that slows or stalls growth and, in extreme cases, causes leaf scorching or premature senescence (Hsiao 1973; Pugnaire et al. 1999; Etzold et al. 2021; Estiarte and Peñuelas 2015). Together, these temperature and soil moisture limitations act as environmental filters, narrowing the period during which growth is possible (Figure 1). Notably, comparable (or even stronger) seasonal constraints occur in seasonally dry forests as is typical in Mediterranean climates, where temperatures may remain suitable for growth year‐round but multi‐month dry seasons impose pronounced water limitation. Across species, leaf flush and renewed growth commonly track the onset of rains and access to plant‐available water (including deep soil moisture), making soil water availability a primary driver of seasonal activity in these systems (Borchert 1994; Ramos et al. 2023; Alvarado et al. 2025).

Light also influences plant growth through two key processes. First, light intensity provides the primary energy source that drives photosynthesis and, consequently, source activity. Second, light carries a wealth of information through its quality and the length of daily light and dark periods (photoperiod), which mediate many physiological processes, including bud set and dormancy transitions (Wang et al. 2024). Both factors may contribute to the common observation that tree growth often peaks near the summer solstice in extra‐tropical regions (Rossi et al. 2006; Etzold et al. 2021; Luo et al. 2018).

## 3 Internal Programming of Plants

Given our relatively robust understanding of environmental influences on plant growth, it may seem straightforward to predict how plants will grow under current and future climatic conditions. Yet, this has proven challenging, particularly when predicting responses under scenarios with extended, climatically favourable growing seasons (Zohner et al. 2021). Here we propose that accurate predictions require incorporating another key, but often overlooked, factor: internal growth control—the genetically encoded developmental program implemented through hormonal and gene‐regulatory networks that may prevent further growth *despite* favourable environmental conditions.

While plants have evolved numerous morphological adaptations to tolerate or avoid harmful environmental conditions, most temperate and boreal woody perennials cope with seasonal fluctuations in temperature and moisture by temporally escaping these conditions. They do this by aligning the timing of their life‐history events (phenology) with a cycle of growth and dormancy that balances survival, reproduction and growth over the long lifespan typical of many tree species (Delpierre et al. 2016). Hence, the phenological sequence can impose internal switches in resource allocation—from vegetative growth to reproduction (flowering, fruit maturation) and storage (Stearns 1989; Chapin et al. 1990). These transitions act as additional internal filters, further narrowing the effective window during which vegetative growth can occur, regardless of environmental potential (Figure 1).

## 4 The Concept of (In)determinate Growth

In annual plants, growth ends with the production of flowers to form fruits and seeds: a signal in the apical meristem triggers a switch in resource investment from vegetative growth to building a reproductive structure with no point of return, signalling the end of the life‐cycle (Poethig 2003; Huijser and Schmid 2011). A shoot axis that terminates in a flower or other modified structure and ceases meristematic activity is considered ‘determinate’ (Barthélémy and Caraglio 2007). In woody perennial species, however, flowers are typically produced on lateral shoots or buds, preserving the main vegetative axis and allowing continued structural expansion. However, ‘determinate growth’ also refers to a temporal suspension of the meristem, which leads to a time lag between the formation of organs and their expansion (Kozlowski 2012; Hallé et al. 1978).

**FIGURE 2:**
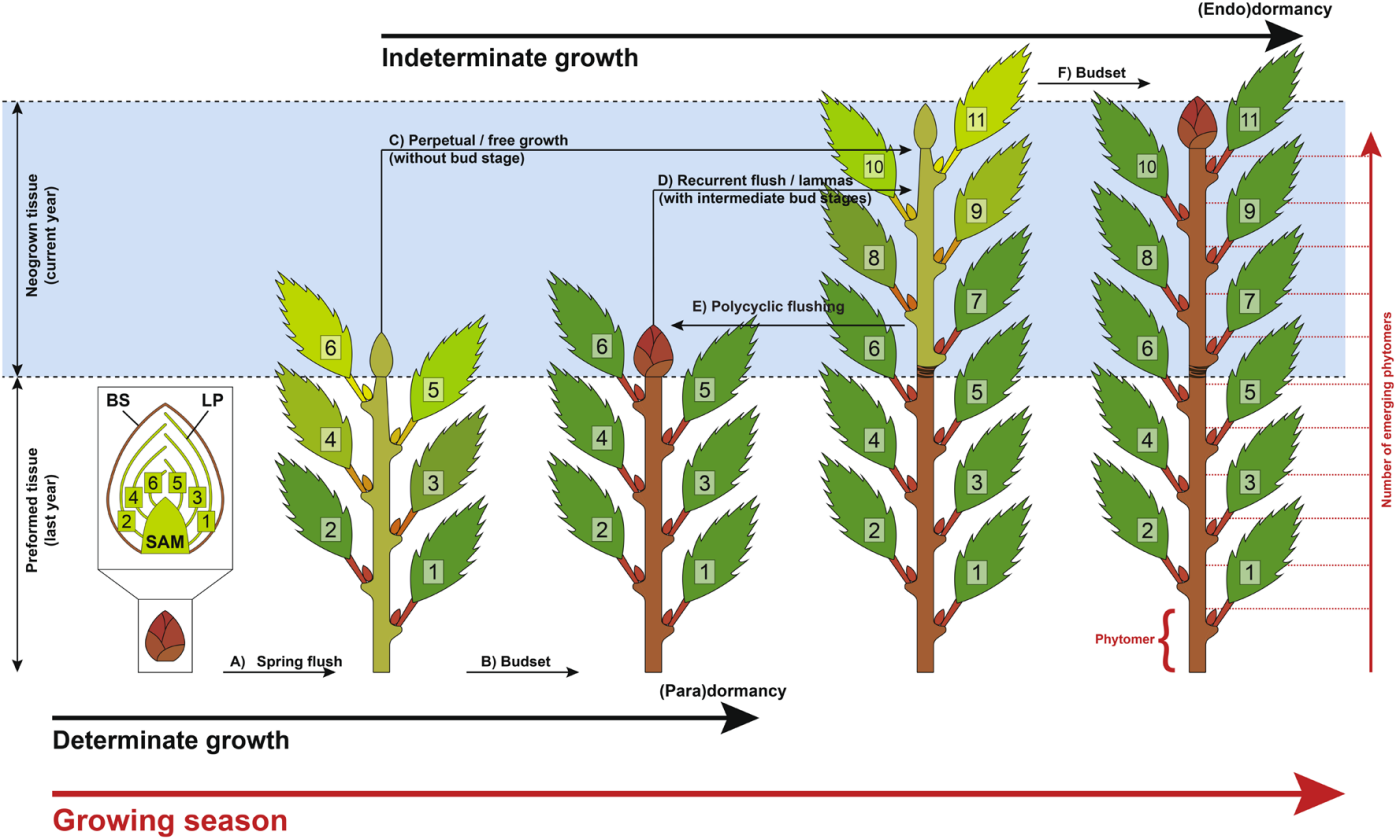
Determinate and indeterminate growth within one growing season for species producing terminal buds. Commonly, all tree species deploy buds during their first (spring) flush from prebuilt and overwintering leaf primordia (LP) protected by bud scales (BS; A). Determinate growing species set buds (B) that are under hormonal suppression to inhibit any further activity of the shoot apical meristem—SAM (paradormancy). The basic unit of a shoot is the phytomer which is composed of a node, a leaf, the axillary bud, and an internode (see bottom right). Indeterminate growing species continue to produce new phytomers directly (C) or through one (D) to several (E) intermediate bud stage(s). Finally, most species set their terminal buds (F) and enter full dormancy (endodormancy).

In their first year, all woody seedlings exhibit indeterminate growth. They begin growth from an embryonic shoot meristem and typically prioritize rapid leaf and canopy establishment, reflecting a juvenile, largely opportunistic phase of development (Borchert 1976). In subsequent years, many temperate and boreal tree species preform their new shoot increments during the previous growing season, overwintering them as primordial shoots with leaves and internodes in hardened buds to be ‘ready‐to‐go’ when spring arrives. Once the leaves flush and the preformed internodes elongate, species differ in how long the shoot apical meristem remains competent to continue organ production (Figure 2). Some species exhibit strongly determinate shoot extension, in which the terminal meristem is intrinsically downregulated soon after the spring flush and remains inactive for the rest of the season, consistent with a prolonged internal suppression that is hormonally mediated (paradormancy; Lang et al. 1987; Figure 4; e.g., *Fraxinus* spp., *Acer* spp. and *Prunus* spp.). At the other extreme, highly indeterminate species maintain an active shoot apical meristem and can continue producing new leaves and internodes on top of the preformed shoot as long as conditions remain favourable, sometimes stretching their indeterminate growth well into autumn (e.g., *Sequoia* spp., *Cupressus* spp. and *Alnus* spp.). Between these extremes, many species occupy an intermediate position: they continue to produce neoformed leaves and internodes after the preformed increment, but growth cessation is often coordinated by seasonal cues—frequently photoperiod acting through hormonal regulation (e.g., *Pinus* spp., *Betula* spp. and *Populus* spp.; Maurya and Bhalerao 2017; Triozzi et al. 2018; Böhlenius et al. 2006). Indeterminate growth can occur either through continuous activity of the shoot apical meristem forming new leaves and internodes without buds (free growth), or through the formation and subsequent flushing of buds mid‐season without a period of dormancy (second flushing or lammas growth; Figure 2).

Although this concept is often presented dichotomously (Kozlowski and Pallardy 1997; Lechowicz 1984), but see (Kikuzawa 1983; Damascos et al. 2005), with species classified as either determinate or indeterminate growers, most species likely exist along a gradient with numerous intermediate forms. For instance, many temperate oaks (*Quercus* spp.) are considered determinate growers, but can exhibit multiple flushes. Similarly, Douglas‐fir ( *Pseudotsuga menziesii* ) gradually adopts a more determinate growth habit with maturity (Borchert 1976; Heuret et al. 2006).

Importantly, the degree of determinacy can be quantified at the species level, at least for the shoot apical meristem as the ratio of leaves produced during the relevant growth period to the number of leaf primordia preformed in the bud, with values above 1 indicating increasing neoformation (Box 1). Figure 4 provides a literature‐based classification of determinacy for some common species, many of which fall into an intermediate position.

BOX 1 **Metrics and proxies related to (in)determinacy.**

To advance our understanding of growth strategies under climate change, we need not only improved methods for quantifying the degree of (in)determinacy—but also empirical approaches that move beyond a simple binary classification. An organ‐level metric at the shoot apical meristem captures the defining features of (in)determinacy and allows it to be explicitly defined and quantified. Temporal dynamics of shoot growth provide a related but indirect proxy that can be applied from individual shoots to crowns and entire forest stands.

*Ratio of preformed to neoformed organs:* The ratio of the number of leaves (or leaf scars) produced during a growing season to the number of leaf primordia preformed in dormant buds provides a direct, mechanistic and quantitative measure of shoot‐level (in)determinacy. Values close to one indicate growth constrained to preformed organs, whereas values greater than one reflect the production of neoformed organs and thus indeterminate development. This ratio should be interpreted relative to the relevant growth period considered. In species with more than one distinct growth phase within a year, estimates may need to integrate all flushes contributing to annual shoot development, or be reported separately for distinct flushes. Because the expression of neoformation depends on environmental conditions, indeterminate species may appear more determinate in short or unfavourable years. Preformation and neoformation have been documented for more than a century (Moore 1909) and several studies have quantified the number of preformed primordia and compared it with the final number of emerged leaves or phytomers (Damascos et al. 2005; Kikuzawa 1983; Guédon et al. 2006). Quantification typically relies on bud dissection, but emerging non‐destructive approaches such as micro–CT imaging offer promising alternatives for assessing bud structure and preformation (Kovaleski et al. 2019). This metric remains the most conceptually robust approach for defining and quantifying the level of (in)determinacy across species.

*Seasonal growth dynamics of the shoot apical meristem:* Temporal patterns of shoot elongation provide a complementary perspective on (in)determinacy at the shoot apical meristem. Across species, elongation may occur over a brief period, extend continuously through the growing season, or appear in multiple distinct growth phases, reflecting differences in how apical meristem activity is maintained or reactivated. Such dynamics can be quantified using repeated shoot measurements or phenological observations and increasingly through high‐resolution terrestrial LiDAR and photogrammetric techniques that capture shoot elongation and structural change at the scale of entire crowns or stands (Zhang et al. 2019; Jin et al. 2022). While temporal growth dynamics do not distinguish preformed from neoformed organs directly, they remain closely linked to shoot apical meristem activity and provide a scalable proxy for the seasonal expression of (in)determinacy. Repeated measurements also allow shoot‐elongation curves to be reconstructed, whose shape differs systematically among species (single, short sigmoid vs. prolonged or multi‐phase extension; Hover et al. (2017); Kuster et al. (2014)). Specifically, curve‐derived parameters such as the duration of shoot expansion relative to the growing season provide a quantitative proxy for the degree of (in)determinacy.

## 5 Evolutionary Trade‐Offs: The Cost of Certainty and Flexibility

In most temperate and boreal ecosystems, determinate and indeterminate growth strategies co‐occur, indicating that neither strategy is universally superior. Instead, they reflect contrasting solutions to a fundamental trade‐off between growth certainty and growth flexibility under seasonal and variable environments.

Indeterminate species are characterized by continued or episodic activity of the shoot apical meristem during the growing season. This does not necessarily imply faster intrinsic growth rates, but rather a greater capacity to extend growth when conditions remain favourable. Such temporal flexibility allows individuals to exploit transient resource availability and recover from partial damage, traits that are advantageous in disturbed or early‐successional environments. Accordingly, indeterminate growth is frequently associated with early successional species (Marks 1975). However, maintaining active meristems later into the season also increases exposure to environmental stressors such as summer drought and early autumn frost, thereby raising the risk of tissue loss or incomplete maturation (Figure 4). As a result, indeterminate growth strategies often involve higher interannual variability in performance and, in some cases, shorter tissue or organismal lifespans (Millet et al. 1999; Brienen et al. 2020).

In contrast, determinate species preform a substantial portion of their shoot growth in advance and deploy this tissue during a restricted period of the growing season. This strategy limits short‐term plasticity, because the year's shoot‐growth potential is set during bud formation in the preceding year and is largely realized by expanding preformed organs (Gordon et al. 2006). At the same time, it reduces exposure of sensitive tissues to late‐season stress and synchronizes growth with the most predictable and favourable part of the season. As a consequence, determinate growth strategies are often associated with stronger local adaptation and reduced sensitivity to short‐term climatic fluctuations (Leites and Benito Garzón 2023).

These contrasting strategies help explain the coexistence of determinate and indeterminate species within the same landscapes. For example, western larch ( *Larix occidentalis* ) and Douglas‐fir ( *Pseudotsuga menziesii* ) frequently co‐occur despite marked differences in growth determinacy. Western larch exhibits more flexible, indeterminate shoot growth and performs well in environments shaped by frequent disturbance, whereas Douglas‐fir shows more determinate growth and stronger local adaptation, conferring stability under predictable seasonal regimes (Roskilly and Aitken 2024). Such differences suggest niche differentiation along gradients of disturbance frequency, environmental stress and climatic predictability, rather than outcomes driven by straightforward competitive dominance.

Together, these patterns indicate that growth determinacy reflects a trade‐off between flexibility and reliability in seasonal environments. Under ongoing climate change—marked by longer growing seasons, greater climatic variability and increasing environmental stress in many regions—it remains uncertain whether determinate or indeterminate growth strategies will be favoured. Resolving both how trees regulate their degree of (in)determinacy and which levels along this trait continuum are advantageous under future conditions will be critical for predicting shifts in species performance, coexistence and forest composition.

## 6 The Performance of (In)determinacy With Climate Change

Spring warming has advanced the onset of leaf emergence by up to a month compared to pre‐industrial times (Vitasse et al. 2022), reflecting an earlier reactivation of shoot apical and cambial meristems under warmer conditions. In contrast, the cessation of growth in autumn has shifted far less than expected given the extension of climatically favourable conditions (Zani et al. 2020; Zohner et al. 2023). As a result, phenological sequences are increasingly observed to shift as a whole toward spring rather than being symmetrically extended at both ends of the growing season (Keenan and Richardson 2015; Meng et al. 2024). This asymmetric response highlights a key constraint relevant to growth (in)determinacy: while growth onset is highly plastic and environmentally responsive, the duration of meristematic activity later in the season appears more tightly regulated by plant internal processes and differs markedly among species. Notably, leaf senescence is often a poor proxy for the cessation of primary growth and under uneven seasonal warming the cessation of shoot growth can even advance while senescence is delayed (Zohner and Renner 2019). Such constraints on growth cessation help explain why longer growing seasons do not necessarily translate into increased growth or biomass production (Zani et al. 2020; Bose et al. 2025; Wolkovich et al. 2025; Rao et al. 2026).

**FIGURE 3:**
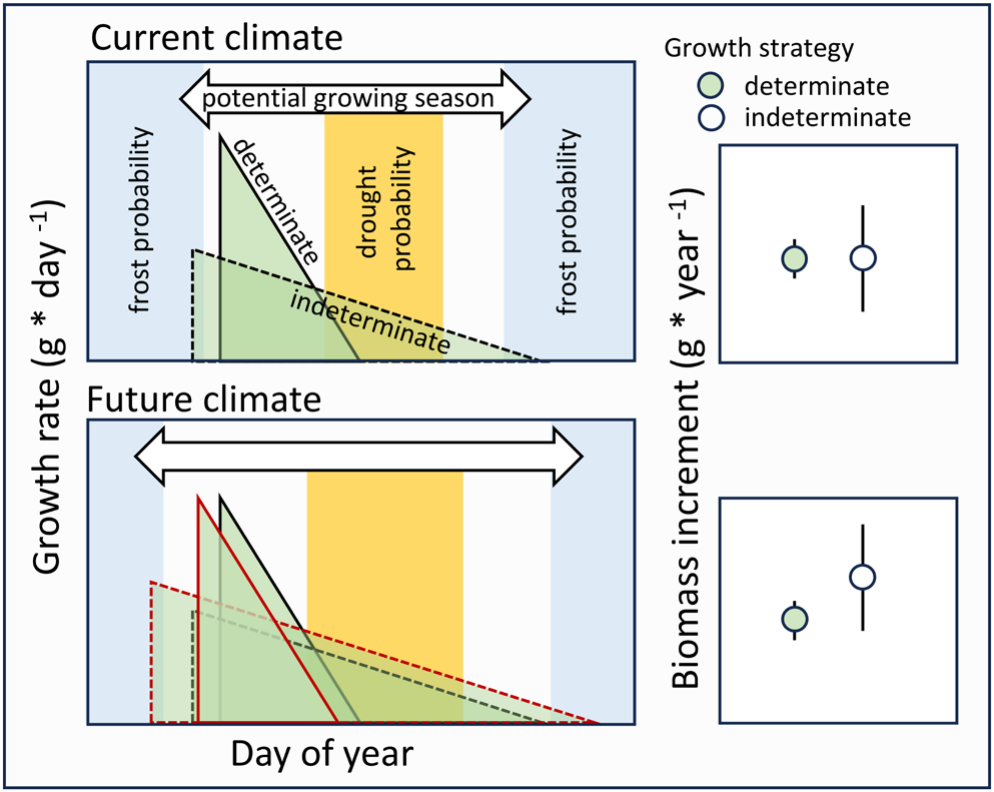
Hypothesized predictions of growth rates under current (upper panels) and future (lower panels) climate for determinate and indeterminate growing species. Note that the indeterminate strategy is more exposed to the risk of frost and drought events while the determinate strategy condenses most growth within a safe period. In the current climate the indeterminate strategy is in balance with benefiting from the full climatic growing season in some years with some drawbacks in other years, resulting in the same mean yearly biomass increment, but with a higher variation (right panel). In a future climate the indeterminate strategy might benefit from longer growing seasons, resulting in an overall higher mean annual biomass increment compared to determinate growers, but this will be dependent on the severity of drought that overlaps the potential growth period of determinate species. Triangle outlines represent the activity window for a current (black) versus a future (red) climate.

Along the continuum of growth determinacy, we hypothesize that species positioned toward the more determinate end will largely maintain their fixed seasonal growth schedules under climate warming, with limited capacity to extend growth later into the season. In regions where climate change primarily increases heat and water stress during mid to late summer, such species are expected to show reduced annual biomass production because their restricted growth window increasingly overlaps with unfavourable conditions (Figure 3).

In contrast, we predict that species toward the indeterminate end of the continuum will be more capable of prolonging meristematic activity into late summer and autumn when conditions become favourable again, potentially resulting in higher annual production under some future climate scenarios (Caffarra and Donnelly 2011; Richardson et al. 2009; Zohner et al. 2020; Figure 3). This capacity for temporal extension of growth is particularly common among early successional species, which tend to leaf out earlier in spring and senesce later in autumn than late‐successional species. Their earlier leaf‐out is consistent with generally lower chilling and forcing requirements for bud burst and dormancy release (Heide 1993; Laube et al. 2014; Hu et al. 2022; Baumgarten et al. 2021; Basler and Körner 2012) and in many cases weaker photoperiod sensitivity (Basler and Körner 2012).

Extreme climatic events and increasing environmental stress provide an important context for evaluating these predictions. More determinate growth strategies concentrate growth earlier in the season and may therefore reduce exposure to unfavourable late‐season conditions by operating within a relatively narrow spring to early‐summer window that often coincides with more favourable temperature and soil moisture conditions (Pearson and D'Orangeville 2022). In systems where drought or heat stress typically intensifies later in the season, this strategy may provide a wider safety margin against late‐season stress, consistent with the ‘late‐summer drought avoidance hypothesis’ (Pearson and D'Orangeville 2022; D'Orangeville et al. 2018).

Where climate change introduces novel or intensified drought stress within this window, however, determinate species are likely to experience reduced annual production and limited capacity for recovery within the same growing season once canopy development has been compromised (Silvestro et al. 2023; Figure 3).

Species with more indeterminate growth habits often remain green and physiologically active for longer periods, leafing out early in spring and senescing late in autumn, with deciduous species occasionally retaining functional foliage until the first freezing events (Figure 3). As a consequence, a substantial fraction of their growth period coincides with parts of the growing season during which drought stress is projected to intensify under future climates, particularly in water‐limited regions (Figure 3). While this extended activity increases exposure to environmental extremes, it also creates opportunities for compensatory growth when conditions become favourable again.

In essence, the flexible growth schedule of indeterminately growing species may allow plants to: (1) produce tissue better adapted to harsh environmental conditions as it is formed in the current season (e.g., smaller leaves with higher specific leaf area) and (2) catch up and compensate later in the season by another production boost (Stevens and Lindroth 2005). Mediterranean ecosystems provide a clear example of this flexibility, where many species suspend growth during summer drought but resume meristematic activity in autumn or winter when conditions allow (Volaire et al. 2023). Evidence that trees can take advantage of such a ‘second growing season’ after summer drought has been found in pines in Mediterranean climates through polycyclic flushing—a form of indeterminate growth (Girard et al. 2011; Figure 3).

We therefore hypothesize that climate change will increasingly disrupt established phenological cycles, favouring species and genotypes that are more plastic in rearranging their activities by resuming growth and/or restoring reserves (e.g., starch, nutrients) later in the year, thereby recovering from and compensating for stress‐induced damage and losses. Such differential capacity to exploit newly available growth opportunities or to recover after stress is expected to intensify competition among co‐occurring species by altering the timing and magnitude of resource uptake, potentially leading to the reassembly of forest communities toward species occupying more indeterminate positions along the growth continuum. This shift may be reinforced if climate change increases not only mean stress but also climatic variability (van der Wiel and Bintanja 2021), because flexible (plastic) growth schedules can capitalize on transient favourable windows and compensate after setbacks (Richter et al. 2012; Chevin et al. 2010; Botero et al. 2015).

## 7 (In)determinacy Beyond Primary Shoot Growth

Although (in)determinacy is most clearly defined *sensu stricto* at the shoot apical meristem, its relevance may extend beyond primary shoot growth because shoot extension and cambial growth are coordinated within the plant. Primary growth governs crown expansion and annual leaf deployment, thereby influencing total leaf area and canopy photosynthetic capacity (Girard et al. 2011; Soolanayakanahally et al. 2015). Primary shoot growth also establishes the framework within which later radial growth can occur by determining the extent and geometry of axes along which cambial activity is expressed. Empirical studies showing close within‐season associations between shoot extension and stem or radial growth therefore point less to simple control by one process over the other than to coordinated development between apical and cambial meristems (Cuny et al. 2012; Zhang et al. 2019).

At the same time, we do not suggest that cambial growth is simply controlled by newly formed leaves or by current assimilate supply. Primary and secondary growth have partially distinct controls and constraints and cause‐and‐effect relationships between them are not straightforward (Hartmann and Trumbore 2016; Trugman and Anderegg 2025; Cabon 2026). Rather, from a whole‐plant perspective, production of additional leaves, xylem and phloem is likely to be coordinated because each is only advantageous if matched by corresponding hydraulic and carbon‐transport demands. In highly indeterminate species, prolonged primary growth may be part of a broader syndrome in which axis extension and cambial activity remain active for longer.

Although vascular development is not preformed in the same mechanistic sense as shoot organs enclosed in buds, vascular tissues can nonetheless show seasonal carry‐over. In particular, where conductive phloem persists through winter, part of the transport system available early in the next season was established previously and may therefore represent a functional analogue of preformation (Savage 2020; Savage and Chuine 2021). The timing and duration of cambial cell division constrain the rate and seasonal extent of secondary growth, while subsequent cell maturation can further restrict wood formation (Lupi et al. 2010).

At the same time, cambial cell production can cease even when environmental conditions remain favourable and the canopy is still photosynthetically active, pointing to internal regulation of secondary growth (Buttò et al. 2020; Arend et al. 2024). Consistent with this, carbon assimilation and wood growth are widely decoupled in time and respond to different climatic drivers, indicating pervasive sink rather than source control of tree growth across biomes (Cabon et al. 2022). In temperate oaks, for example, current‐year radial growth is insensitive to climate variability after midsummer even though roughly a third of annual gross primary productivity is fixed during this late‐season period, with wood formation ceasing well before photosynthesis declines (Rao et al. 2026).

In contrast to shoot and cambial growth, below‐ground meristems appear to follow a more opportunistic strategy. Root apical meristems can remain active whenever local conditions permit, with rhizotron studies documenting root growth during winter under suitable soil temperatures (Lyford and Wilson 1966), suggesting that roots often lack a clearly defined dormant phase (Radville et al. 2016; Marchand et al. 2025). However, the extent to which below‐ground growth reflects direct environmental control, coordination with above‐ground growth, or genetically constrained strategies remains unresolved (Abramoff and Finzi 2015; Makoto et al. 2020; Silvestro et al. 2025; Campioli et al. 2024).

Together, these observations suggest that although (in)determinacy is most rigorously defined at the shoot apical meristem, shoot‐level variation may still provide a useful lens for studying coordination among plant growth processes and their consequences for whole‐plant performance.

## 8 Future Directions

A central challenge emerging from this perspective is to better understand how variation along the continuum of growth (in)determinacy shapes tree performance under changing climatic conditions. Rather than representing discrete strategies, degrees of determinacy define bounded trait spaces that differ among species but can vary with age, provenance and environmental context. While the degree of determinacy is largely constrained at the species level, evidence from provenance trials shows meaningful intraspecific variation; for example, populations from lower latitudes often exhibit higher frequencies of second flushes than those from northern origins (Rudolph 1964). Clarifying how these species‐specific bounds and population‐level adjustments interact with warming, drought and increasing climatic variability will be critical for understanding how forest communities respond and reassemble under climate change. Extending this framework beyond woody perennials to include herbaceous species and perennial grasses may further help test the generality of (in)determinacy as a developmental constraint on growth timing, while highlighting differences arising from life form and the absence of secondary growth.

One key knowledge gap concerns how different degrees of (in)determinacy help trees avoid or buffer environmental stress while still maintaining the capacity to resume growth, repair tissues, replenish reserves and compensate for earlier losses within the same season. Resolving the trade‐off between escaping unfavourable conditions and exploiting longer or intermittently favourable growing periods is essential for anticipating how forest communities will change over the coming decades.

**FIGURE 4:**
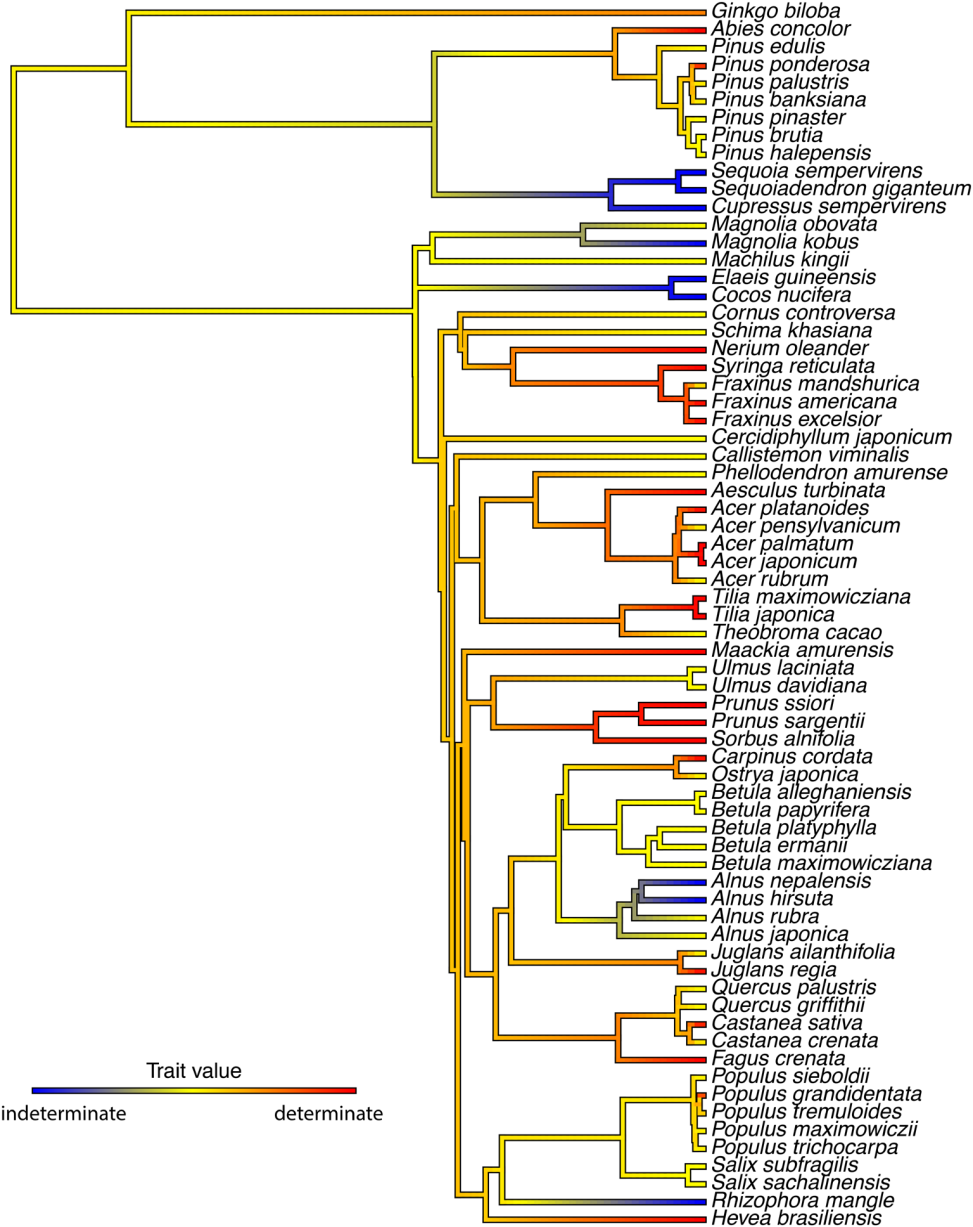
Phylogenetic tree of plant species coloured by growth determinacy trait values, visually representing the evolutionary distribution of growth strategies across taxa. This tree includes species for which shoot growth determinacy data were compiled (mainly from Hallé et al. (1978) and Kikuzawa (1983)), with the phylogeny pruned from the comprehensive seed plant tree of Smith and Brown (2018) to match the trait dataset. Growth determinacy was scored as indeterminate (blue), intermediate (yellow) and determinate (red), with mean values calculated per species when multiple entries existed. These values were then mapped as a continuous trait onto the phylogeny using the contMap() function from the phytools R package.

Understanding whether shoot‐level (in)determinacy is associated with analogous patterns of developmental carry‐over or seasonal flexibility in other organs may provide insight into the evolution of this trait, especially through cross‐species analyses. Both growth forms seem to occur across most species of the same family or clade and hence appear to have evolved repeatedly in different groups (Figure 4), making it difficult to speculate on which strategy is ancestral (but see Hariharan et al. 2016) and how rapidly (in)determinacy can evolve. As data on the degree of (in)determinacy across species accumulates (see Box 1), such analyses could potentially provide insights into this and also aid forecasting for unsampled species.

Finally, future work should aim to link the temporal dynamics of primary (shoot and root) and secondary (cambial) meristems with patterns of reserve storage and reproductive allocation. Integrating shoot increment formation, wood production, non‐structural carbohydrate dynamics and reproductive effort within a common temporal framework will be essential for understanding whole‐tree carbon allocation. Correlating annual tree‐ring formation with shoot increments—while accounting for the timing of inter‐annual phytomer production and distinguishing between preformed and neoformed tissues (Figure 2)—offers a promising path toward such integration and toward improving mechanistic predictions of tree growth and forest carbon dynamics under future climates.

## Author Contributions

Frederik Baumgarten and EM Wolkovich initiated this project. Frederik Baumgarten took a lead in drafting, designed figures, and performed phylogenetic analyses. All authors contributed substantially to the writing of the article.

## Funding

This work was supported by Schweizerischer Nationalfonds zur Förderung der Wissenschaftlichen Forschung (P500PB_210943).

## Conflicts of Interest

The authors declare no conflicts of interest.

## Data Availability

The data supporting the phylogeny are archived in the following public repository: https://doi.org/10.5281/zenodo.18637216.
